# The Potential Benefits of Red Beetroot Supplementation in Health and Disease

**DOI:** 10.3390/nu7042801

**Published:** 2015-04-14

**Authors:** Tom Clifford, Glyn Howatson, Daniel J. West, Emma J. Stevenson

**Affiliations:** 1 Department of Sport, Exercise and Rehabilitation, Faculty of Health and Life Sciences, Northumberland Building, Northumbria University, Newcastle upon Tyne, NE1 8ST, UK; E-Mails: tom.clifford@northumbria.ac.uk (T.C.); glyn.howatson@northumbria.ac.uk (G.H.); d.j.west@northumbria.ac.uk (D.J.W.); 2 Water Research Group, School of Environmental Sciences and Development, Northwest University, Potchefstroom 2520, South Africa

**Keywords:** beetroot, betalains, nitrate, antioxidants, inflammation, oxidative stress

## Abstract

In recent years there has been a growing interest in the biological activity of red beetroot (*Beta vulgaris rubra*) and its potential utility as a health promoting and disease preventing functional food. As a source of nitrate, beetroot ingestion provides a natural means of increasing *in vivo* nitric oxide (NO) availability and has emerged as a potential strategy to prevent and manage pathologies associated with diminished NO bioavailability, notably hypertension and endothelial function. Beetroot is also being considered as a promising therapeutic treatment in a range of clinical pathologies associated with oxidative stress and inflammation. Its constituents, most notably the betalain pigments, display potent antioxidant, anti-inflammatory and chemo-preventive activity *in vitro* and *in vivo*. The purpose of this review is to discuss beetroot’s biological activity and to evaluate evidence from studies that specifically investigated the effect of beetroot supplementation on inflammation, oxidative stress, cognition and endothelial function.

## 1. Introduction

The well-documented health benefits of a diet high in fruit and vegetables has led to a growing interest in so-called “functional foods” and their application in health and disease. In recent years, the root vegetable *Beta vulgaris rubra*, otherwise known as red beetroot (herein referred to as beetroot) has attracted much attention as a health promoting functional food. While scientific interest in beetroot has only gained momentum in the past few decades, reports of its use as a natural medicine date back to Roman times [1]. Today, beetroot is grown in many countries worldwide, is regularly consumed as part of the normal diet, and commonly used in manufacturing as a food colouring agent known as E162 [2,3].

The recent interest in beetroot has been primarily driven by the discovery that sources of dietary nitrate may have important implications for managing cardiovascular health [4]. However, beetroot is rich in several other bioactive compounds that may provide health benefits, particularly for disorders characterised by chronic inflammation. Consequently, the potential role for beetroot as an adjunct treatment in several clinical conditions will be presented; Specifically, the aims of this review are twofold: (1) to highlight evidence from recent studies showing the physiological and biological actions of beetroot; and (2) to evaluate its use as a nutritional intervention in health and disease, with a special emphasis on experimental studies relating to oxidative stress, inflammation, endothelial function and cognition.

Recent studies have provided compelling evidence that beetroot ingestion offers beneficial physiological effects that may translate to improved clinical outcomes for several pathologies, such as; hypertension, atherosclerosis, type 2 diabetes and dementia [1,5,6,7,8]. Hypertension in particular has been the target of many therapeutic interventions and there are numerous studies that show beetroot, delivered acutely as a juice supplement [9,10,11] or in bread [12,13] significantly reduce systolic and diastolic blood pressure. Further discussion of beetroot’s anti-hypertensive potential is summarised in several reviews: [14,15,16].

Beetroot’s effect on the vasculature is largely attributed to its high inorganic nitrate content (250 mg∙kg^−1^ of fresh weight; [17]). Nitrate itself is not considered to mediate any specific physiological function; rather, nitrates beneficial effects are attributed to its *in vivo* reduction to nitric oxide (NO), a multifarious messenger molecule with important vascular and metabolic functions [14,18]. The generation of NO via nitrate involves a series of sequential steps that have been well described in the literature [4,19]. Briefly, ingested nitrate is first absorbed through the upper part of the small intestine into the systemic circulation [4,15]. It is then estimated that 25% of the circulating nitrate enters the entero-salivary cycle where bacterial species located at the posterior aspect of the tongue bioactivate or reduce salivary nitrate to nitrite [16,19]. Because salivary bacteria facilitate the reduction reaction that converts nitrate to nitrite, spitting out saliva or taking oral anti-bacterial treatments, like dental mouthwash for example, has been shown to diminish nitrate-nitrite conversion [10,18]. Under normal circumstances, however, salivary nitrite is re-absorbed into the circulation via the stomach where it is metabolised to NO and other nitrogen oxides by a variety of reductase enzymes [4,10,13].

**Figure 1:**
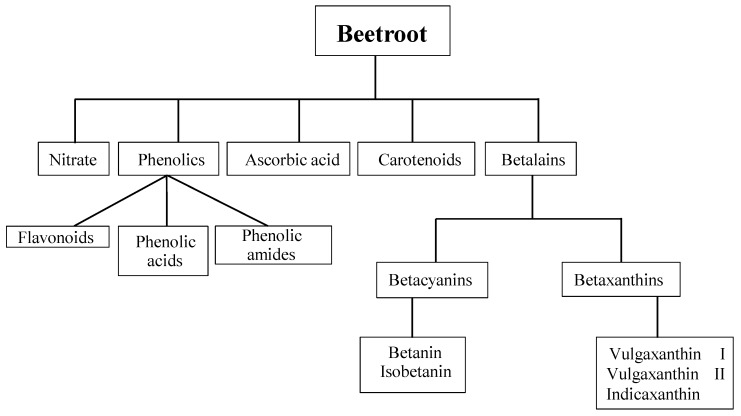
Overview of potentially bioactive compounds in beetroot (based on data from [1,2,20]).

However, as previously mentioned, nitrate is not the only constituent of beetroot proposed to have beneficial effects in health and disease. Beetroot is a rich source of phytochemical compounds (Figure 1), that includes ascorbic acid, carotenoids, phenolic acids and flavonoids [2,20,21]. Beetroot is also one of the few vegetables that contain a group of highly bioactive pigments known as betalains [22,23]. Members of the betalain family are categorised as either betacyanin pigments that are red-violet in colour or betaxanthin pigments that are yellow-orange in colour [1]. A number of investigations have reported betalains to have high antioxidant and anti-inflammatory capabilities *in vitro* and a variety of *in vivo* animal models [3,23,24,25,26]. This has sparked interest in a possible role for beetroot in clinical pathologies characterised by oxidative stress and chronic inflammation such as liver disease [1,23], arthritis [27] and even cancer [28,29,30,31].

## 2. Bioavailability 

For a food component to be considered beneficial for health it must be bioavailable *in vivo*, that is, following ingestion, the active compounds are absorbed through the gastro-intestinal tract and made available in the circulation, in sufficient quantities, to be utilized by cells [21,32]. However, in order to reach the systemic circulation and exert any salubrious functions, a food component must maintain its molecular structure through several phases of digestion that each present a significant metabolic challenge for the molecule and affect its eventual rate and extent of absorption [33,34]. It is therefore critically important that any alleged health benefit of a food source be firstly verified with well-designed bioavailability studies that characterise the extent of its *in vivo* absorption [34]. In this respect, the bioavailability of both inorganic nitrate and the betalains, the major bioactive components of beetroot, have been considered in the literature. The high bioavailability of inorganic dietary nitrate is well established and there are reports of close to 100% absorption following digestion [35]. The extent to which betalains are absorbed is, however, less clear. 

Two studies have directly investigated betalain bioavailability by measuring their appearance in human urine after ingesting a single bolus of beetroot juice [36,37]. Kanner *et al.* [37] identified 0.5%–0.9% of the ingested betacyanins (betanin and isobetanin) in volunteer’s urine in the 12 h after consuming 300 mL of beetroot juice. This indicates that although in small amounts, betacyanins can be successfully absorbed in humans. They also showed that the peak urinary elimination rate of betacyanains (indicative of absorption), occurred 2–4 h after ingestion; however, there was a high level of inter-individual variability within this time period. Frank *et al.* [36] reported similar findings while investigating betacyanin bioavailability. After providing six healthy participants with 500 mL of beetroot juice, they identified betacyanins in urine at concentrations equivalent to ~0.3% of the ingested dose over a 24 h period. These studies might be interpreted to suggest only small level of bioavailability; however, it is important to realise that betacyanins are unlikely to be exclusively eliminated via the renal pathway [36] Indeed, the use of urinary excretion as a sole indicator of bioavailability has received criticism because it does not account for the biliary and circulatory clearance of compounds, thus underestimating true bioavailability [33]. In addition, the extent to which betalains are metabolised and structurally transformed to secondary metabolites is yet to be characterized, but should be taken into consideration when examining their bioavailability [36].

Given these limitations, Tesoriere *et al.* [38] employed a different approach to investigate the bioavailability of betalains. Tesoriere and colleagues developed a simulated *in vitro* model of the human intestinal epithelium using Caco-2 cell monolayers to mimic a functional barrier. This model allowed them to examine whether betalains can be absorbed through a functioning intestinal barrier and hence give an indication of their bioavailability. They demonstrated that two betalains; betanin and to a greater extent indicaxanthin were well absorbed through the simulated model of the intestinal lining (Caco-2 cell monolayer) and mostly in their unmetabolised form via paracellular transport. The latter finding is particularly important, because it reveals that betalains can be absorbed into the systemic circulation in their unchanged form, allowing them to retain their molecular structure and high biological activity [34]. There was some evidence that betanin may be absorbed through transcellular transport as well. Nevertheless, it is important to note that results from *in vitro* experiments, even when designed to mimic the biological milieu of the human GI tract, do not necessarily translate *in vivo*, given that several other factors (*i.e.*, first pass metabolism, interactions with gut microflora and protease enzyme degradation) have a significant influence on the concentration of the nutrient that eventually reaches the circulation [33,34].

In addition to the betalain family, other aforementioned plant derived antioxidants have been identified in beetroot, including epicatechin, rutin, and caffeic acid [2], which to varying degrees appear to be well absorbed and bioavailable in humans [39]. Although, the bioavailability of these compounds and other phenolics from beetroot have not been individually determined, there are data describing the bioavailability of the total phenolic compounds present in beetroot. Netzel *et al.* [40] measured the urinary excretion of total phenolic substances following a single 500 mL bolus of beetroot juice. They identified ~685 mg of phenolic compounds in participant’s urine ≤24 h following beetroot juice ingestion; 97% more than the ~347 mg identified after consuming water (*i.e.*, basal concentrations). While the relative bioavailability from the individual compounds could not be determined, these findings clearly show that beetroots phenolic constituents are extremely well absorbed and likely increase beetroot’s *in vivo* antioxidant power. 

Taken together, the results of the aforementioned studies provide a good base of evidence that beetroot is a bioavailable source of bioactive compounds in humans. With that said, further work is still required to firstly; elucidate the bioavailability of beetroot’s individual bioactive components and secondly; to establish the extent that plasma, biliary and other metabolic pathways contribute to the excretion of these components. Together, these data would give a better understanding of beetroots phytochemical bioavailability and thus elucidate the potential as a health-promoting intervention for humans.

## 3. Oxidative Stress

Beetroot supplementation might serve as a useful strategy to strengthen endogenous antioxidant defences, helping to protect cellular components from oxidative damage. Under normal metabolic conditions, the biological environment of a cell is considered to be in a state of redox balance, or in other words, an equilibrium exists between reducing (antioxidants) and oxidising (pro-oxidants) agents [41,42]. Molecules capable of oxidation are commonly known as reactive oxygen and nitrogen species (RONS) and are continuously generated in cellular metabolism [42]. At these low concentrations, RONS play an important role in a diverse multitude of cellular and biochemical processes, including gene expression, cell proliferation, apoptosis and muscular contraction [41,42,43,44]. However, excess exposure of a cell to exogenously generated RONS (UV radiation, xenobiotics) or endogenously synthesised RONS (aberrant cell metabolism, inflammation), can overwhelm the cells antioxidant defences, causing an imbalance in redox homeostasis, which gives rise to the condition typically referred to as oxidative stress [42]. This imbalance may overwhelm the endogenous antioxidant defence network leaving DNA, carbohydrate, protein and lipid structures susceptible to oxidation and functional impairments [45,46,47].

In some instances, cells can suffer from acute spells of oxidative stress that temporarily weaken antioxidant defences [48]. This can occur through excessive heat exposure, infectious pathogens and strenuous physical exercise, which are capable of generating RONS that leave cells vulnerable to transient oxidation [44,48]. However, in many human disease states, such as cancer, oxidative stress is a chronic disorder perpetuated by continual and excess production of RONS that induce long-term cellular disruption [48,49]. A previous estimate [42] suggested that oxidative stress plays a role in the pathophysiology of over 200 clinical conditions. It is therefore unsurprising that many antioxidant food sources have been evaluated for their ability to scavenge RONS and avert oxidative stress.

Beetroot is as an exceptionally rich source of antioxidant compounds. The betalain pigments in particular, has been shown by several *in vitro* studies to protect cellular components from oxidative injury [37,50,51]. For example, in the study by Kanner *et al.* [37] two betalain metabolites (betanin and betanidin) were shown to reduce linoleate damage induced by cytochrome C oxidase and lipid membrane oxidation induced by H_2_O_2_-activated metmyoglobin and free iron (AA-Fe). The authors also reported that betanin, the most abundant betalain found in beetroot (300–600 mg∙kg^−1^), was the most effective inhibitor of lipid peroxidation. Betanin’s high antioxidant activity appeared to stem from its exceptional electron donating capacity and ability to defuse highly reactive radicals targeting cell membranes [37]. However, as alluded to earlier, betalains are not the only antioxidant compounds present in beetroot. Beetroot contains several highly bioactive phenolics, such as rutin, epicatechin and caffeic acid which are also known to be excellent antioxidants [2,36,39]. Furthermore, nitrite and other NO donors akin to beetroot have been shown to suppress radical formation and directly scavenge potentially damaging RONS such as superoxide and hydrogen peroxide, suggesting nitrate may also exhibit antioxidant effects [19,52,53].

**Figure 2:**
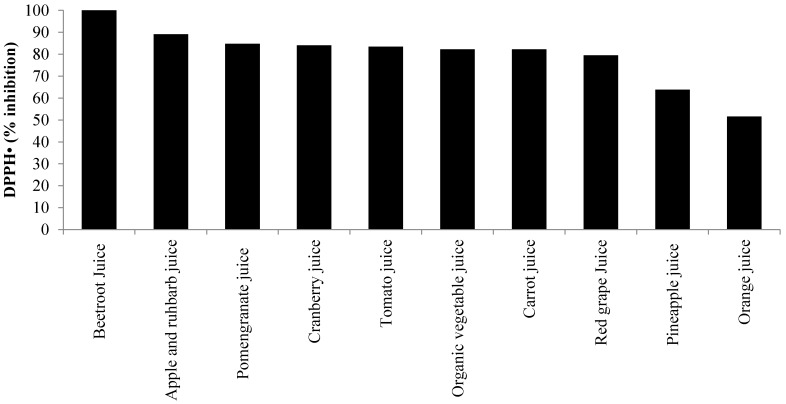
A comparison of the 2,2-diphenyl-1-picrylhydrazyl (DPPH•) inhibiting capacity (%) exhibited by 10 popular fruit and vegetable beverages available in the UK (values based on data from [56,57]).

**Figure 3:**
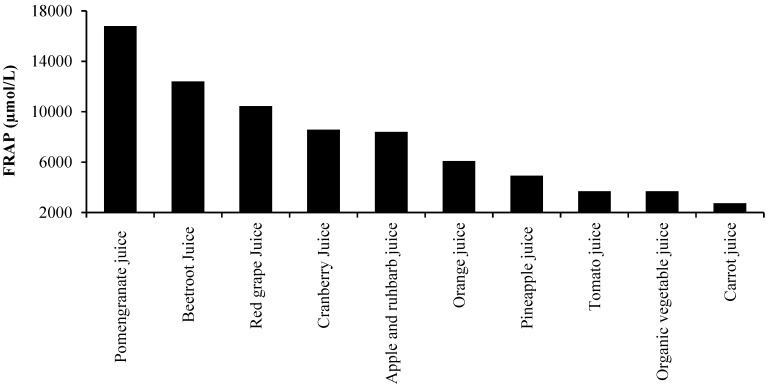
The free radical antioxidant power (FRAP) of 10 commercially available fruit and vegetable beverages post a simulated *in vitro* model of human digestion (values based on data from [56,57]).

A number of studies report that beetroot, in the form of a juice supplement, protects against oxidative damage to DNA, lipid and protein structures *in vitro* [27,54,55]. A study by Wootton-Beard and Colleagues suggests that a key mechanism by which beetroot juice exerts its antioxidant effects is by scavenging radical species [56]. They found that two commercially available beetroot juices inhibited *in vitro* radical formation in the 2,2-diphenyl-1-picrylhydrazyl (DPPH•) and (3-ethylbenzothiazoline-6-sulfonicacid) ABTS• assays by 100% and 92%, respectively. Importantly, when these assays were repeated, but in conditions designed to simulate the human digestive process, the juices still retained ≥55% of their pre-digestion radical scavenging capacity. Furthermore, the antioxidant capacity of both drinks, as measured by ferric reducing antioxidant power (FRAP), was higher than the other 22 vegetable juice drinks under investigation. In another study [21], they showed that the FRAP of beetroot juice actually increases following simulated digestion. This is probably a consequence of several compounds being structurally altered to secondary metabolites that possess antioxidant functions [21,56]. Further work from this group has shown that the antioxidant capacity of beetroot juice is comparable to or higher than a variety of fruit and vegetable juices (See Figure 2 and Figure 3) [56,57]. Interestingly, the antioxidant capacity of beetroot juice in both the (DPPH•) and FRAP assays was far greater than more well-known vegetable juices, such as tomato and carrot, and fruit juices, such as orange and pineapple, with only pomegranate juice displaying a higher antioxidant capacity in the FRAP assay.

In addition to being a source of antioxidants *in vitro*, a growing body of evidence using animal models illustrates that beetroot exhibits radical scavenging ability *in vivo* (see Table 1). In a recent study [23], rats were provided with 1–3 mL∙kg∙bm^−1^ of a beetroot pomace extract for 7 days prior to being exposed to 2 mL∙kg∙bm^−1^ of carbon-tetrachloride (CCl4), a well-established carcinogen and RONS generator. After CCI4 administration, liver homogenate was removed from rats pre-treated with the beetroot extracts and those acting as controls (*i.e.*, CCl4 only). Rats treated with beetroot extracts expressed significantly lower levels of lipid peroxidation measured as thiobarbituric acid reactive substances (TBARS). Furthermore, the beetroot extracts appeared to maintain endogenous antioxidant activity (reduced glutathione, glutathione peroxidase and catalase enzymes) at normal cellular concentrations following the oxidative insult. This led the authors to speculate that in response to *in vivo* cellular attack, beetroot may exhibit indirect antioxidant effects that act to up regulate antioxidant defence mechanisms [23].

Similar antioxidant effects have also been reported with studies using beetroot juice. Providing rats beetroot juice (8 mL∙kg∙bm∙day^−1^) for 28 days was shown to attenuate lipid peroxidation, protein oxidation and DNA damage following xenobiotic induced liver injury [54]. In a more recent, rats were fed beetroot juice (8 mL∙kg∙bm∙day^−1^ for 28 days) and treated with the carcinogen 7,12-dimethylbenz[a]anthracene (DMBA) on day 27 and 28 of the beetroot juice-feeding period study [58]. Several markers of liver damage and inflammation were significantly increased following the DMBA treatment; however, these were markedly reduced in the rats pre-treated with beetroot juice compared to the control group that received water only (see Table 1). There were no differences in DNA damage between the groups. Intriguingly, rats given beetroot juice only (*i.e.*, not treated with DMBA) exhibited increased activity of phase II detoxifying enzymes (GST and NQO1), which play an important role in endogenous antioxidant defense.

The enhanced endogenous antioxidant activity *in vivo*, by beetroot is a consistent finding in the literature (see Table 1). According to recent *in vitro* data, such effects might be related, in part, to betanin and its effect on signalling pathways that mediate the transcription of antioxidant genes. Esatbeyoglu *et al.* [59] found that betanin (extracted from beetroot) dose dependently (5–15 μm) increased the activity of nuclear factor (erythroid-derived 2)-like 2 (Nrf2), a transcription factor that activates a gene promoter sequence: the antioxidant response element (ARE) responsible for the transcription of several endogenous antioxidant enzymes [59,60,61]. Krajka-Kuźniak *et al.* [62] presented similar findings, showing that betanin (2, 10 and 20 μm concentration) activates the Nrf2-ARE binding sequence in non-tumor human hepatic cell lines. Furthermore, this led to increased activity and mRNA expression of several phase II detoxifying enzymes, including glutathione *S*-tranferases and NAD(P)H:quinone oxidoreductase, which play important roles in host defense against xenobiotics. This raises the possibility that beetroot’s antioxidant potential is not limited to just scavenging and suppressing RONS, but includes the ability to reinforce the endogenous antioxidant network; however, whether such effects translate *in vivo*, particularly in humans is yet to be investigated.

Given these findings, it should also be considered that other compounds in beetroot (and their downstream metabolites upon ingestion) possess similar effects to betanin on transcriptional activity. Thus, *in vivo*, these compounds and metabolites could work synergistically to activate the NRF2-ARE pathway, which, in turn, mediates an increase in endogenous antioxidant activity. Such a possibility deserves further attention. 

**Table 1:** Overview of human and animal studies investigating the effects of beetroot and its derivatives on oxidative stress and inflammation.

Authors	Cohort under Investigation	Dosage	Antioxidant Capacity of Treatment	Duration	Toxic inducing Protocol	Inflammation	Oxidative Stress	Enzymatic Antioxidant Activity
[54]	48 male wistar rats	Beetroot juice delivered by gavage (8 mL∙kg∙bm∙day^−1^; ≈1.92 mL∙day^−1^)	23.5 μmol Trolox equivalents∙mL^−1^	28 days	Intraperitoneal injection of CCl_4_ (2 mL∙kg∙bm^−1^) or NDEA (150 mL∙kg∙bm^−1^)	N/A	TBARS ↓ PC ↓ DNA damage ↓	SOD ↑ GPX ↑ CAT ↑ GR ↑
[63]	80 male ICR mice	Betalains from beetroot delivered orally (0, 5, 20 and 80 mg∙kg∙bm∙day^−1^; ≈0–1.44 mg∙day^−1^)	N/A	30 days	Exposed to cobalt-60-γ-gamma radiation (6.0 Gy, 1.5 Gy min^−1^)	N/A	MDA ↓	SOD ↑ CAT ↑ GSH ↑
[64]	24 male wistar rats	Beetroot juice delivered by gavage (8 mL∙kg∙bm∙day^−1^)	N/A	28 days	Intraperitoneal injection of NDEA (150 mL∙kg∙bm^−1^)	LDH ↓ AST ↓ ALT ↓	DNA damage ↓	GST ↑
[27]	10 osteoarthritic patients	Capsules made from beetroot extract delivered orally (70–200 mg∙day^−1^)	N/A	10 days	N/A	TNF-α ↓ IL-6 ↓ RANTES ↓ GRO-α ↓	AOPP ↓	N/A
[23]	48 albino wistar rats	Beetroot pomace extract delivered intraperitoneally (1–3 mL∙kg∙bm∙day^−1^; ≈0.2–0.6 ml∙day^−1^)	N/A	7 days	Intraperitoneal injection of CCl_4_ (2 mL∙kg∙bm^−1^)	N/A	MDA ↓	GSH ↑ GSHPx ↑ GR ↑ CAT ↑
[65]	24 albino wistar rats	Extracts of fresh beetroot delivered orally (250 and 500 mg∙kg∙bm∙day^−1^; ≈45–90 mg∙day^−1^)	90.1% radical inhibition in the DPPH• assay (500 μg∙mL^−1^)	28 days	Intraperitoneal injection of gentamicin (8 mg∙kg∙bm^−1^ for 8 days)	IL-6 ↓ TNF-α ↓ MPO ↓ NF-κB ↓	MDA ↓	CAT ↑ NP-SH ↑
[58]	24 female Sprague-dawley rats	Beetroot juice delivered by gavage (8 mL∙kg∙bm∙day^−1^; ≈1.92 mL∙day^−1^)	N/A	28 days	Intraperitoneal injection of DMBA (10 mg∙kg∙bm^−1^ for 2 days)	LDH ↓ ALT↓	N/A	GST ↑ NQO1 ↑

NDEA, *N*-nitrosodiethylamine; GST, glutathione *S*-transferase; RANTES, regulated upon activation normal T cell growth; GRO-α, regulated oncogene-alpha; CCl_4_ carbon tetrachloride; GSH, reduced glutathione; GSHPx, glutathione peroxidase; GR, glutathione reductase; GPX, glutathione peroxidase; TNF-α, tumour necrosis factor-alpha; TBARS, thiobarbituric acid reactive species; PC, protein carbonyls; SOD, superoxide dismutase; ICR, imprinting control region; MDA, Malondialdehyde; CAT, catalase; AOPP, advanced oxidation protein products; IL-6, interleukin-6. Gy, gray unit; LDH, lactate dehydrogenase; ALT, alanine aminotransferase; AST, aspartate aminotransferase; MPO, myeloperoxidase. NF-κB, nuclear factor kappa B; NP-SH, non-protein sulfhydryl; DMBA, 7,12-dimethylbenz[a]anthracene; NQO1, NAD(P)H dehydrogenase [quinone] 1.

Collectively, the studies to date provide evidence that beetroot is an excellent source of antioxidants that protects cellular components from oxidation *in vitro* and importantly, *in vivo*. This would suggest that beetroot supplementation might be a promising adjunct strategy to help manage diseased states propagated by oxidative stress, such as liver injury and cancer. With that said, there is still a lack of well-conducted human trials, which precludes any definitive recommendations for its clinical use. Moreover, data is currently limited to paradigms inducing oxidative stress through exogenous pathways (*i.e.*, xenobiotics) while endogenously generated RONS play a significant role in human disease [66]. In this respect, strenuous physical exercise could serve as a useful model to study the antioxidant potential of beetroot and its constituents in humans. There are several reports documenting that the mechanical and metabolic muscle damage sustained during intense exercise induces short-term oxidative stress, which can persist for several days until redox balance is restored [67,68]. Therefore, it should be considered that application of a beetroot preparation following an exercise task might serve as a useful model to give an insight into beetroot’s efficacy as an antioxidant agent in humans, and most importantly, provide a more in-depth understanding of its potential application in clinical settings. 

## 4. Inflammation 

Under normal circumstances, inflammation is regarded as a beneficial process, governing our innate response to biological or physical stimuli such as trauma, infection and other pathogens that may cause the organism harm and disrupt homeostasis [69,70,71]. With that said, immune activation may still have undesirable consequences for the host. In the short term, redness, swelling, pain and diminished function may be experienced at the site of inflammation; however, more concerning is the potential long-term implications if inflammation persists, and is unresolved [71,72]. Failure to remove the invading element and restore normal immune function can cause chronic inflammation resulting in long-term cell dysfunction [73]. Chronic inflammation is often implicated in the onset and progression of several clinical disorders such as obesity, liver disease, cancer and heart disease [69,74,75]. 

Since the 1970’s, traditional treatment for inflammatory disorders has been non-steroidal anti-inflammatory drugs (NSAIDS) [71]. However, these drugs, particularly in chronic doses, may actually have deleterious consequences for health and evoke negative side effects [74,76]. Above all, they have been deemed ineffective in the treatment of many inflammatory related conditions [74]. Consequently, focus has shifted towards the anti-inflammatory effects of natural food sources and their potential use as alternatives to synthetic NSAID treatments [72]. 

**Figure 4:**
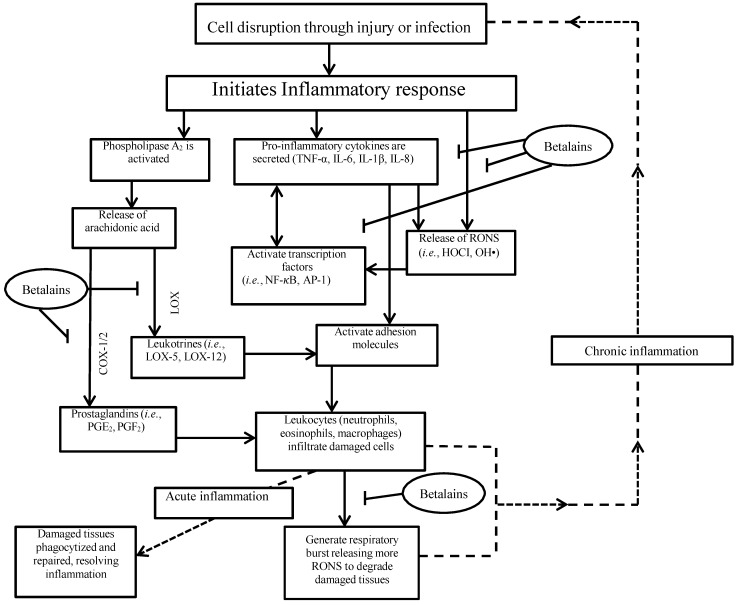
Illustration of the inflammatory cascade in response to cellular attack and possible pathways where betalains may exhibit inhibitory effects. PGF2, Prostaglandin F2; PGE2, Prostaglandin E2; COX ½, Cyclooxygenase 1 and 2; LOX, lipoxygenase; LOX-5, 5-lipoxygenase; LOX-12, 12- lipoxygenase; HOCI, Hypochlorous acid; OH•, Hydroxyl radical; NF-κB, Nuclear Factor-Kappa B; AP-1, Activator protein 1; IL-6, Interleukin-6; IL-8, Interleukin-8; IL-1β, Interleukin-1 beta; TNF-α, tumour necrosis factor-alpha.

Betalains and beetroot extracts have emerged as potent anti-inflammatory agents. At least part of their anti-inflammatory effects seems to be mediated by interfering with pro-inflammatory signalling cascades and are summarised in Figure 4. The most important of these is the Nuclear Factor-Kappa B (NF-κB) cascade, as it directly activates and transcribes most gene targets that regulate and amplify the inflammatory response (*i.e.*, cytokines, chemokines, apoptotic and phagocytic cells) [43]. Consequently, NF-κB activity plays a central role in the inflammatory processes that manifest in chronic disease [43]. In a recent study [65], NF-κB DNA-binding activity was dose-dependently attenuated in nephrotoxic rats administered a beetroot extract for 28 days (250 mg or 500 mg∙kg∙bm^−1^). Furthermore, kidney homogenates from the beetroot treated rats had lower concentrations of immune cells (TNF-α, IL-6 and MPO) and reduced signs of oxidative damage (MDA), which could be directly related to the blunting of the NF-κB pathway. These effects are likely to be mediated, at least in part, by the betalains present in beetroot; recent evidence shows that betanin treatment (25 and 100 mg∙kg∙bm^−1^ for 5 days) significantly inhibits NF-κB DNA-binding activity in rats induced with acute renal damage [77]. Betalains have also been shown to markedly supress cyclooxygenase-2 (COX-2) expression *in vitro*, which is an important precursor molecule for pro-inflammatory arachidonic acid metabolites known as prostaglandins [3,26,50]. Reddy *et al.* [50] who found that betanin (IC50 value 100 μg·mL^−1^) inhibited cyclooxygenase-2 (COX-2) enzyme activity by 97%, first illustrated this. It is interesting to note that although a slightly higher concentration of betanin was required, its COX-2 inhibitory effects were comparable or greater than several phenolic compounds (cyanidin-3-O-glucoside, lycopene, chlorophyll, b-carotene, and bixin) and anti-inflammatory drugs (Ibuprofen, Vioxx and Celebrex). This raises the possibility that betanin rich beetroot supplements, in sufficient doses, could exhibit anti-inflammatory effects to rival synthetic drugs.

A recent study from Vidal *et al.* [26] provided further support for the anti-inflammatory effects of betalains. As well as supressing COX-2 synthesis, betanidin, extracted from beetroot, dose dependently inhibited (to 9% of control activity), lipoxygenase (LOX), a catalytic enzyme vital for the synthesis of pro-inflammatory leukotriene molecules [70]. Interestingly, these inhibitory effects appeared to be mediated by a blocking action on membrane binding activity, indicating that betalains target cell signalling pathways at the molecular level, acting in a similar fashion to selective COX-2 inhibitor drugs [26,70].

There are a limited number of studies demonstrating that beetroot supplements have anti-inflammatory effects *in vivo*. Pietrzkowski *et al.* [27] showed that therapeutic administration of betalain-rich oral capsules made from beetroot extracts alleviated inflammation and pain in osteoarthritic patients. After 10 days of supplementation (100, 70 or 35 mg per day), the pro-inflammatory cytokines; tumour necrosis factor-alpha (TNF-α) and interleukin-6 (IL-6), had decreased from baseline by 8.3%–35% and 22%–28.3%, respectively. The activity of two chemokines; regulated oncogene-alpha (GRO-alpha) and regulated upon activation normal T cell growth (RANTES) were also markedly inhibited by the beetroot treatment. Furthermore, the moderated inflammatory response coincided with a significant reduction in self-reported pain on the McGill Pain Questionnaire. Krajka-Kuźniak *et al.* [64] examined the protective effect of beetroot juice (8 mL∙kg∙bm∙day^−1^ for 28 days) on markers of liver injury and inflammation induced by the toxic chemical N-nitrosodiethylamine (NDEA) in rats. Compared to an untreated control, the beetroot juice conferred significant hepatic protection against a range of inflammatory markers induced by NDEA administration; lactate dehydrogenase, aspartate aminotransferase, gamma glutamyl transferase and alanine aminotransferase were all shown to be markedly attenuated. In a more recent study, El Gamal *et al.* [65] fed rats either water (control) or oral doses of beetroot ethanol extract (250 or 500 mg∙kg∙bm∙day^−1^) for 28 days; from day 20–28 they were treated with the nephrotoxic drug gentamicin (85 mg∙kg∙bm∙day^−1^). After 28 days, kidney homogenates were removed from both groups and analysed for several markers of renal damage. They found that the beetroot-treated rats had significantly lower concentrations of several pro-inflammatory mediators, including Il-6, TNF-α, myeloperoxidase (representing neutrophil infiltration) and the transcription factor NF-κB. They also found reduced oxidative stress (MDA, uric acid) and increased endogenous antioxidant activity (CAT, non protein sulfhydryl; NP-SH) in the homogenates removed from the rats pre-treated with beetroot. Taken together, these studies support the notion that beetroot supplementation offers anti-inflammatory protection *in vivo*; however, well designed, long-term clinical trials are clearly required to elucidate whether such a strategy would assist in the management of inflammatory-related disorders.

The antioxidant and anti-inflammatory activity of beetroot has also led to interest in its potential use in diseases characterized by aberrant immune cell function. Indeed, chronic inflammation is increasingly being implicated in the development of malignant tumours and evidence is accumulating to suggest betalain extracts obtained from beetroot may suppress these effects. For instance, Lechener *et al.* [78] examined whether long term (35 weeks) treatment with a betacyanin containing extract (78 μg∙mL∙day^−1^ of E162, red food colour prepared from beetroot) would inhibit tumour incidence in rats exposed to a potent tumour promoter (*N*-nitrosomethylbenzylamine). In comparison to control, the beetroot extracts markedly inhibited cell proliferation, angiogenesis and tumorgenesis in oesophageal lesions, effects largely attributed to its radical scavenging and anti-inflammatory activity. This was evidenced by the significantly reduced number of inflammatory lymphocytes present in the oesophageal tumours of the beetroot-treated rats only. The chemo-preventive effects of betacyanin extracts have also been observed in lung, skin and liver cancer cells in animal models, and recently, in human prostate, skin, breast and pancreatic tumour cells [28,29,30,31]. Furthermore, co-administration of beetroot extracts with doxorubicin, an effective but highly toxic chemotherapy drug, significantly reduces its cytotoxicity, probably by modulating the drug’s induction of tumour promoting RONS [28,31]. Although far from conclusive, these initial findings in animal and human cell lines suggest beetroot supplementation holds promise as a future strategy to at least help manage some of the symptoms of inflammation in cancer. 

## 5. Endothelial Function 

As described earlier, nitrate delivered via a beetroot source is metabolised to nitrite, which can be further reduced to produce NO [4,13]. The conversion of nitrite to NO can be catalysed by a number of molecules with reductase potential (*i.e.*, electron donors), and to date, several proteins (*i.e.*, deoxymyoglobin, xanthine oxioreducatse) and antioxidants (*i.e.*, vitamin C) have been reported to facilitate this reduction [4,79,80]. One of the most important functions of endogenous NO is to maintain endothelial function [13,81]. The endothelium plays a critical role in the regulation of vascular homeostasis by maintaining thrombotic activity, platelet function, vascular tone and the delicate balance between the release of vasodilating (*i.e.*, NO, prostacyclin) and vasoconstricting agents (*i.e.*, endothelin-1, thromboxane) [81,82]. Because NO mediates many of the endothelium’s functions, a depletion in NO availability, as seen with aging, has been singled out as the principal cause of endothelial dysfunction [81]. Endothelial dysfunction is proposed as a primary risk factor for several cardiovascular disorders and has been implicated in the pathogenesis of hypertension and atherosclerosis [16,83]. Therefore, beetroot, as a natural NO donor, has been explored as a nutritional approach to preserve or restore endothelial function. 

Webb *et al.* [10] were the first to investigate the effects of a beetroot supplement on endothelial function in healthy participants. They measured brachial artery (BA) endothelial function using the flow mediated dilation technique (FMD), which involved calculating BA dilation before and after a 20 min ischemic insult. The ischemic procedure (BA occlusion) was effective at inducing endothelial dysfunction, as evidenced by the 60% decrease from pre to post BAFMD response. However, when participants were pre-treated 2 h prior with a single serving of beetroot juice (500 mL; 23 mmol of nitrate) the BAFMD response was maintained at pre-ischemic levels, suggesting that beetroot juice acted to preserve endothelial function.

Hobbs *et al.* [12] extended these findings, examining the acute intake of a novel beetroot enriched bread (100 g beetroot, nitrate; 1.1 mmol) on micro vascular function and peripheral arterial stiffness in young healthy males. Although arterial stiffness, assessed by pulse wave velocity and augmentation index, was unaffected by the intervention, the beetroot bread increased micro vascular vasodilation, as measured by changes in cutaneous perfusion using laser doppler imaging (LDI). Endothelium-independent vasodilation (perfusion units) was ~343% greater in the 6 h after ingesting the beetroot enriched bread compared to the control bread. Importantly, this study provided evidence that even a small nitrate load (1.1 mmol) can augment marked improvements in intravascular function. Similar vascular effects were also reported in a study with older populations. Using apparently healthy but slightly obese, older participants (~61 years), Joris and Mensink, [83] investigated whether beetroot juice supplementation would prevent postprandial impairments in BAFMD. In a randomized crossover design, BAFMD response fell by ~1.6% in the control condition, whereas after beetroot juice (140 mL, nitrate; 500 mg) the impairment was only ~0.4%, indicative of a beetroot-mediated protective effect on postprandial endothelial function.

Replicating the aforementioned findings in volunteers with an increased risk of endothelial dysfunction has proved more difficult. For instance, Kenjale *et al.* [84] reported that acute beetroot juice intake (500 mL) had no influence on endothelial function in peripheral arterial disease (PAD) patients, as assessed by BAFMD response (% arterial dilation) to a hyperaemic stimulus. In another study, type 2 diabetic volunteers were given either beetroot juice (250 mL·day^−1^; nitrate: 7.5 mmol) or a nitrate depleted placebo but otherwise nutritionally matched beetroot juice for 14 days [85]. After the treatment period, BAFMD response was similar between control and beetroot juice groups (4.94% *vs.* 4.97% change in vessel diameter, respectively) and no differences in micro vascular vasodilation, as measured by LDI, could be detected between the two conditions. However, perhaps the lack of an effect in these studies is not surprising, given that unlike the studies with healthy cohorts, most volunteers were already receiving vasoactive medications for their respective conditions. It is conceivable that these medications diminished or confounded any potential vascular response afforded by nitrate. Perhaps a more profound response to beetroot supplementation could be elicited in pre-clinical patients, *i.e.*, those not yet requiring prescription medications that interfere with vascular function. An alternative explanation for the findings in the latter study is that bioactive constituents (*i.e.*, betalains, caffeic acid) other than nitrate (likely present in both the nitrate depleted placebo and nitrate rich beetroot juice) could have mediated a dilatory response. Indeed, there is evidence that other antioxidants such as flavonoids possess dilatrory effects in humans [86]. If betalains or indeed other antioxidants present in beetroot exerted similar effects in this study then BAFMD and micro vascular function would not be expected to differ between the two conditions. However, the dilatory effects of these compounds and consequences for endothelial function are yet to be investigated and therefore such an effect can only be speculated at present. 

It is also important to note that several disease states, including type 2 diabetes, are characterised by persistent inflammation and an excess production of RONS [87,88]. This could limit the efficacy of nitrate supplementation, because RONS such as superoxide (O_2_-) directly react with NO, diminishing its bioavailability [85]. Therefore, any benefits of the surplus NO generated by ingesting dietary nitrate could be reduced in the presence of oxidative stress. To counter this, perhaps these pathologies would benefit from higher doses of beetroot juice, not only to increase the amount of ingested nitrate (and NO generation) but also to provide a stronger antioxidant defence (*i.e.*, betalains) against RONS. Such an approach might help enhance nitrate mediated NO bioavailability. Further research is required to establish the role of beetroot supplementation in endothelial dysfunction. 

## 6. Cognitive Function

Cognitive function deteriorates with age and one of the key pathological events that precedes its development is reduced cerebral blood flow [89,90,91]. Indeed, an age related decrease or impairment in cerebral perfusion has been implicated in several neurological disorders associated with poor cognitive ability, such as brain damage, clinical dementia and Alzheimer’s disease [92,93]. One of the major triggers and risk factors for the onset and development of cerebral hypo-perfusion is a disruption in neurovascular function; an effect that is, in part, mediated by impaired NO activity [92,93]. A diminished capacity to generate NO can impair the normal function of cerebral energy metabolism (*i.e.*, glucose delivery) and neuronal activity (*i.e.*, cellular communication), which over a chronic period might induce neurodegeneration and cognitive deficits [90,91,92]. Therefore, it is conceivable that a NO generator like beetroot has the potential to improve cerebrovascular blood flow and challenge detriments in cognitive function.

Two recent human studies examined the influence of dietary nitrate on cerebral blood flow. Presley *et al.* [6] measured cerebral perfusion after providing a group of older adults (~75 years) a high nitrate diet (~12 mmol) including beetroot juice, or a nitrate depleted diet (~0.09 mmol) for 24 h. Magnetic resonance imaging (MRI) revealed that the high nitrate diet stimulated a substantial and preferential increase in frontal cortex perfusion, a region of the brain responsible for essential cognitive processes such as executive function, working memory and task-switching. Further work by Bond *et al.* [89] supports these conclusions, showing a decrease in cerebrovascular arterial resistance (indicative of increased cerebral blood flow) following a single serving of nitrate rich beetroot juice (500 mL). However, it is important to note that the subjects in this study were young (~21 years), asymptomatic and apparently disease-free, limiting the application of these findings in elderly and diseased populations. 

Although long-term clinical trials are yet to be conducted, two recent preliminary studies explored the influence of acute beetroot supplementation on age-related cognitive function. In one of these studies, older (~67 years), type 2 diabetics, supplemented with 250 mL of beetroot juice (nitrate: 7.5 mmol) for 14 days, experienced a significant improvement in simple reaction time compared to a control group [5]. However, no effects were evident in other cognitive tests associated with decision-making, rapid processing, shape and spatial memory [5]. Another study from the same group [94] investigated the effects of a beetroot juice supplement (140 mL·day^−1^: nitrate: 9.6 mmol) on cognitive function in healthy, older adults (~63 years). After 3 days of supplementation, they failed to detect any changes in cognitive performance for concentration, memory, attention and information processing ability between the beetroot juice and control condition. Furthermore, a range of brain metabolites associated with neuronal functioning (*N*-acetylaspartate, creatine, choline and myo-Inositol) were not, as hypothesized, upregulated after taking beetroot juice. The somewhat contrasting results between these two studies may be partly explained by differences in participant cohort (type 2 diabetics *vs.* healthy older adults), cognitive tests employed (Kelly *et al.* [94] did not use a simple reaction time test) and dose duration. With regards to the latter posit, perhaps the cerebrovascular response required to elicit measurable changes in cognitive function can only be achieved with longer term dosing strategies that have the potential to induce sustained modifications to cerebrovascular function [94]. Nonetheless, the beetroot-mediated increases in simple reaction time reported by Gilchrist *et al.* [5] is worthy of further investigation, given the potential benefits for clinical populations.

A recent addition to the literature investigated whether acute beetroot juice supplementation could augment cerebral oxygenation status and subsequently aid cognition during a fatiguing exercise task [95]. Beetroot juice (500 mL, nitrate: 5 mmol) ingested 90 min before exercise reduced cerebral deoxygenation status, as measured by near infrared spectroscopy, but failed to improve reaction time and information processing at rest, while cycling at 50%, 70% and 90% of VO_2_ peak and upon completion of the exercise task. As with the previously mentioned study [94], a single serving of beetroot juice may not have been sufficient to induce measurable changes in cognitive performance. This is the only study that has examined beetroot in this fashion; consequently, there is scope for further research to investigate the potential role of beetroot juice in cognitive function at rest and during exercise. 

## 7. Conclusions

Based on the available data, beetroot appears to be a powerful dietary source of health promoting agents that holds potential as therapeutic treatment for several pathological disorders. The powerful antioxidant, anti-inflammatory and vascular-protective effects offered by beetroot and its constituents have been clearly demonstrated by several *in vitro* and *in vivo* human and animal studies; hence its increasing popularity as a nutritional approach to help manage cardiovascular disease and cancer. In the human studies to date, beetroot supplementation has been reported to reduce blood pressure, attenuate inflammation, avert oxidative stress, preserve endothelial function and restore cerebrovascular haemodynamics. Furthermore, although beyond the scope of this review, several studies have now established beetroot supplementation as an effective means of enhancing athletic performance [96,97]. 

## 8. Future Directions 

While the precise mechanisms by which beetroot exerts these beneficial effects are yet to be fully elucidated, the present status quo dictates that the cardio-protective, physiological and metabolic effects are mediated by nitrate and its subsequent conversion to NO, while the anti-oxidative and anti-inflammatory effects are mediated by betalains and other phenolics. However, it is important to recognise that the relative contribution of each compound is far more complex and that additive and synergistic effects cannot be ruled out. 

The present data indicates that the bioactive constituents in beetroot, especially nitrate, appear to be well absorbed and bioavailable in humans. While an optimal dosing strategy does not currently exist, the available data suggests that the multitude of beneficial health effects offered by beetroot can be realised with amounts easily achievable in the diet or with a supplement such as beetroot juice; although at present, there is insufficient evidence regarding the efficacy, and above all, safety of beetroot supplementation to recommend a long-term strategy. Indeed, potentially detrimental health effects arising from excessive intake of beetroot and supplemental derivatives have not been adequately explored. The historical belief that nitrite is a carcinogenic substance in humans still reverberates and concerns have been raised over the uncontrolled use or excessive intake of nitrate rich substances [98,99]. However, natural beetroot juice supplements, as opposed to sodium nitrite salts for instance, are unlikely to pose a significant health risk, at least in the short term [99,100]. While there is presently no anticipated negative health outcomes associated with other constituents of beetroot, consumers should be aware that some supplements (*i.e.*, juices) could have a relatively high sugar content, which might need to be taken into consideration by some individuals (*i.e.*, diabetics). Future studies are still required to evaluate the long term safety of a dietary beetroot intervention particularly in clinical settings. In this respect, beetroot supplementation could be easily administered, and of course, there would be economic and practical benefits of such an approach.

A variety of beetroot based supplements including juices and capsules are now widely available and relatively inexpensive, particularly in comparison to synthetically manufactured drugs. It is therefore critical that future studies focus on the long-term effects (≥4 weeks) of beetroot supplementation.

Although the results so far are promising, most studies tend to use healthy cohorts as participants which limit the applicability of their findings to clinical populations. Furthermore, the overwhelming majority of human studies have focused on the ergogenic or cardio-protective effects of beetroot supplementation, while little attention has been given to the potential anti-oxidative and anti-inflammatory effects. Consequently, there is a great deal of scope to explore the influence of beetroot supplementation in human disorders characterised by chronic inflammation and oxidative stress (*i.e.*, cancers, arthritis, inflammatory bowel disease *etc.*).

Nevertheless, beetroot supplementation is a new and exciting area of research that to date has been shown to induce favourable effects in several facets of health and disease. This indicates that beetroot supplementation holds promise as an economic, practical and importantly natural dietary intervention in clinical settings. Because of beetroot’s high biological activity, there are still several unexplored areas in which supplementation might confer health benefits. This includes but is not limited to; pain reduction, cognitive function, vascular function, insulin resistance, cancer and inflammation, especially in older and diseased populations.
